# Editorial: Novel Approaches to the Neuropharmacology of Mood Disorders

**DOI:** 10.3389/fphar.2019.00589

**Published:** 2019-05-17

**Authors:** Hector J. Caruncho, Lisa E. Kalynchuk, Maria I. Loza, Jose M. Olivares

**Affiliations:** ^1^Division of Medical Sciences, University of Victoria, Victoria, BC, Canada; ^2^Department of Pharmacology, University of Santiago de Compostela, Santiago de Compostela, Spain; ^3^Department of Psychiatry, Alvaro Cunqueiro Hospital, Vigo, Spain

**Keywords:** antidepressant (AD), mood stabilizers, major depression (MDD), bipolar disorder, biomarker

These are exciting times for research on the pharmacology of mood disorders. The “omics” revolutions, the development of noninvasive neuroimaging techniques, the definition of biomarkers, and studies in animal models have all partially redefined the field and contributed to the boom in research articles focusing on novel approaches to the neuropharmacology of both major depressive disorder and bipolar disorder. Recent studies on the fast antidepressant effects of ketamine have also brought this topic to the mainline press, where the effectiveness (or lack of) of psychopharmacological drugs targeting mood disorders has been discussed multiple times.

Although management of mood disorders does not solely imply the use of a psychopharmacological approach, the prescription of antidepressants and mood stabilizers represents the mainstay of the standard of care for the treatment of mood disorders.

This research topic is a collection of reviews and original research articles that focus on the study of mechanistic approaches to decipher specific actions of currently used drugs, and on evaluating possible therapeutic interventions by acting on novel pharmacological targets and analyzing signal transduction pathways that may be involved in mediating the effects of drugs acting on those targets. The topic also collects a series of articles devoted to the analysis of different biomarkers that could be used to predict the therapeutic efficacy of specific pharmacologic treatments particularly in the case of treatment-resistant depression.

The effects of inflammatory processes in mood and anxiety disorders and the possible efficacy of current and novel psychopharmacological approaches in tackling mood disorders symptoms by acting on peripheral and/or central inflammatory events are evaluated in three contributions to the present topic: First, Zhang et al. study the effects of the dual serotonin and norepinephrine reuptake inhibitor venlafaxine in reversing the deficits in cognition and depressive-like behavior induced by cuprisone treatment in rodents, by attenuating demyelination and neuroinflammation. Then, review by Brymer et al. examines the putative antidepressant mechanisms of anti-inflammatory drugs that target tumor necrosis factor alpha (TNFα) and explains how both peripheral and central anti-inflammatory mechanisms may be operative in fostering the antidepressant effects of these drugs. Finally, Nisbett and Pinna contribute an opinion article on how fostering the function of the peroxisome proliferator-activated receptor alpha (PPARα) brings about a decrease in proinflammatory cytokines, and focus on the effects of cannabinoids on PPARα in the context of posttraumatic stress disorder (PTSD). These three contributions emphasize how the anti-inflammatory effects of current antidepressants, like venlafaxine, or of anti-inflammatory drugs, like etanercept [an antagonist of tumor necrosis factor alpha (TNFα)], that have been shown to exert antidepressant effects, or those related to novel targets, like PPARα, may be essential for their therapeutic effects on mood disorders and underline how understanding the mechanistic implications of inflammatory processes in mood disorders may give some clues both to better understand the neurobiology of these disorders and to develop novel and more efficacious drugs.

Another two contributions center on the analysis of circuit and/or molecular mechanisms that can relate to the therapeutic actions of psychopharmacological interventions on mood and anxiety disorders: An original research article by Zhang et al. describes how overexpression in the hippocampal dentate gyrus of the translocator protein of 18 kDa results in anxiolytic effects in an animal model of PTSD and discusses the roles of hippocampal neurogenesis in the formation and maintenance of emotional memories that also pertain to the neurobiology of major depression and bipolar disorder, as it has been proposed in multiple occasions that rescuing of hippocampal neurogenesis may be a mechanism by which antidepressant drugs may reverse some key symptoms in depression. A second original report, authored by Park et al., investigates the actions of liraglutide (a glucogen-like peptide 1 receptor agonist) on mammalian target of rapamycin (mTOR)-mediated signal transduction pathways and on α-amino-3-hydroxy-5-methyl-4-isoxazolepropionic acid (AMPA) receptor activity in hippocampal cell cultures treated with dexamethasone, and its impact on brain-derived neurotrophic factor (BDNF) expression, dendritic outgrowth, and spine formation, which results of clear interest when considering that the fast antidepressant actions of ketamine appear to be based on its effects on all these factors.

A review by Senese et al. recapitulates their results on the direct effects of antidepressant drugs on regulating specific components of G-protein coupled receptors systems, particularly their effects on reversing the increase in G protein coupled receptor (GPCR) subunit localization and clustering into lipid-raft microdomains observed in depression, which they propose are essential for the elucidation of the antidepressant effects of current antidepressant drugs. Interestingly, an original report by Romay-Tallon et al. also analyzes the clustering of GPCRs and other proteins in the plasma membrane of lymphocytes in the repeated-corticosterone model of depression, and shows how analysis of this clustering patterns resembles those observed in depression patients and could be considered as putative biomarkers of therapeutic efficacy in major depression, an aspect that is further discussed and analyzed in a review article by Caruncho et al. These three contributions thereby point toward the importance of the patterns on membrane proteins distribution within specific membrane domains as putative key issues in the neurobiology of depression, and foster the design of additional studies on membrane protein clustering not only to develop novel biomarkers but also as potential drug targets.

The last two contributions to the topic describe original research on biomarkers of therapeutic efficacy for treatment-resistant depression: Veldic et al. describe a pharmacogenomic approach to the analysis of cytochrome P450 2C19 variants in relation to their metabolizer phenotype in treatment-resistant depression and evaluate the implications of their results in terms of differential diagnosis between major depression and bipolar disorder, as well as their implications for pharmacological therapeutics in depression. Finally, Shalbaf et al. evaluate how non-linear entropy analysis of electroencephalography (EEG) can be developed as a biomarker to predict the therapeutic response to repetitive transcranial magnetic evaluation in treatment-resistant depression. Their results indicate the interest of additional EEG studies to validate this approach and thereby contribute to a better management of the use of nonpharmacological therapeutic strategies for treatment-resistant depression.

Overall, the contributions to this topic present a wide-scope and multidisciplinary approach that is essential when evaluating novel pharmacological strategies for the treatment of mood disorders.

## Author Contributions

HC wrote the editorial. LK, ML, and JO read and approved the text.

## Conflict of Interest Statement

The authors declare that the research was conducted in the absence of any commercial or financial relationships that could be construed as a potential conflict of interest.

